# Longitudinal NMR Based Serum Metabolomics to Track the Potential Serum Biomarkers of Septic Shock

**DOI:** 10.7150/ntno.79394

**Published:** 2023-01-01

**Authors:** Swarnima Pandey, Afzal Azim, Neeraj Sinha

**Affiliations:** 1 Centre of Biomedical Research, SGPGIMS Campus, Raebareli Road, Lucknow, 226014, India.; 2 Department of Critical Care Medicine, Sanjay Gandhi Postgraduate Institute of Medical Sciences, Lucknow - 226014, India.

## Abstract

**Background:** Septic shock, with a prolonged hospital stay, has the highest mortality rate worldwide. There is a need for better management of the disease, which requires time-dependent analysis of alteration occurring in the disease condition and subsequent planning of treatment strategies to curb mortality.

**Objective:** The study aims to identify early metabolic signatures associated with septic shock before treatment and post-treatment. It also entails the progression of patients towards recovery, which clinicians could use to analyze treatment efficacy.

**Methods:** The study was performed on 157 serum samples of patients with septic shock. We performed metabolomic, univariate, and multivariate statistics to identify the significant metabolite signature of patients prior to treatment and during treatment by collecting serum samples on the day I, day III, and day V of treatment.

**Results:** We identified metabotypes of patients before treatment and post-treatment. The study showed time-dependent metabolite alteration in ketone bodies, amino acids, choline, and NAG in patients undergoing treatment.

**Conclusion:** This study illustrates the metabolite's journey in septic shock and during treatment, which may be of prospective assistance to clinicians to monitor therapeutics.

## Introduction

Sepsis is a complex pathophysiological syndrome caused due to infection, which exponentially escalates from a localized immune response to a systemic immune response 1. These systemic immune responses result in hyper inflammation, damaging organs like the liver, kidney, lungs, and cardiovascular system, known as a septic shock 2,3. Septic shock, a severe form of sepsis, is a leading cause of death in intensive care units 4,5. In 2017, 11 million sepsis-related deaths were reported, accounting for 19.7% of global deaths 6. Early prognosis, diagnosis, and severity determination are quite challenging in septic shock due to their heterogeneous nature.

The clinicians use various biomarkers like PCT 6 and lactate 7 to evaluate the disease severity and progression. However, their sensitivity and specificity are not enough for understanding this heterogeneous disease 7,8. Sepsis and septic shock manifests in the form of metabolic acidosis 9. Metabolic alteration is essential in providing an insight into the development and response to sepsis 10. These metabolites could be used as biomarkers of sepsis and septic shock 11-13. Numerous studies have already reported metabolic biomarkers of septic and septic shock 13-19; 14-21; 22-26. Better management of sepsis and septic shock is the hour need to curb the mortality rate 21,27. One way could be a regular evaluation of the clinical course and treatment efficacy by repeated metabolic measurements.

Metabolomics is an omics science that is a large-scale analysis of small metabolites, products of metabolism, within a cell, tissue, and body fluids 28,29. Metabolites are the molecular phenotypes that can be used as fingerprints left by a pathway. Nuclear magnetic resonance (NMR) spectroscopy is high throughput quantitative means to perform metabolomics 30,31. This study employs NMR-based metabolomics to study the progression of septic shock.

This study aims to investigate the time course of metabolic events, from prior to treatment to post-treatment. We performed a follow-up on day I, day III, and day V post-treatment commencement to understand the metabolic profile of patients undergoing recovery. Furthermore, our intent was to identify 'metabolic biosignatures' that correlated with patient recovery that clinicians could use. We opted for metabonomics to find insights into the metabolic fingerprint of disease during its recovery. The study aided in generating a 'metabolite dairy' and allowed one to follow each metabolite prior to treatment until its progression to recovery. We also explored the metabolic trend of metabolites in survivors and non-survivors of septic shock.

## Materials and Methods

### Study Design

This study was at first approved by the ethical committee of SGPGIMS and CBMR.

The human serum samples for the study were collected in the intensive care unit of Sanjay Gandhi Postgraduate Institute of Medical Sciences (SGPGIMS), and NMR experiments and data analysis were performed in the Centre for Biomedical Research (CBMR), Lucknow, India. Well-informed written consent was obtained from the patients with septic shock or next to their kin enrolled in the study.

### Patient details

All patients admitted to the ICU, meeting the criteria for sepsis and septic shock according to the Sepsis 3 definition, were included in the study. The study group's serum samples were collected from adults (>18 years of age) with septic shock pre-treatment and post-treatment serum samples. Post-treatment follow-up serum was collected on the day I, day III, and day V of their treatment. There were 157 serum samples of patients with septic shock. Of 157 serum samples, 60 were pretreatment serum samples, and 97 were post-treatment serum samples. Table 2 tabulates all the clinical characteristics of patients in study groups.

### Sample Collection and Preparations

A total of 2ml venous blood samples were collected in a sterile vacutainer and allowed to clot for 30 minutes at room temperature, followed by centrifugation at 2,000 x g for 10 minutes to separate the clot from serum. The serum was transferred into storage vials, frozen, and stored at -80°C until NMR measurements were performed.

Before NMR analysis, samples were thawed for 30 min at 4 °C, vortexed, and subsequently centrifuged for 5 min at 1,500 x g. The aliquots of 250 μl of serum samples were mixed with 250μl of saline buffer solutions (in 100% D2O, NaCl 0.9%, 50 mM sodium phosphate buffer, and pH 7.4) in 5 mm NMR tubes with a co-axial insert containing an external standard compound TSP (Sodium salt of 3-trimethylsilyl-(2,2,3,3-d4)-propionic acid).

### NMR Spectroscopy of serum

The NMR experiments were performed on Bruker Avance III 800 MHz NMR spectrometer having the cryogenically cooled triple-resonance TCI, with a 5 mm broadband inverse probe-head and Z-shielded Gradient at 298 K. The acquisition parameter used for 1D CPMG using Carr-Purcell-Meiboom-Gill pulse sequence (cpmgpr1d, standard Bruker pulse program) included spectral sweep width: 20.55, relaxation delay: 5 s, no of scans: 128, echo time of 200 μs repeated 300 times with line broadening of 0.3Hz and an exponential window function collected into 64k data points.

### Data Processing

The spectra were manually phased and baseline corrected. The recorded spectra were binned into integrated spectral buckets of 0.001 ppm for CPMG spectra using Bruker AMIX software (Version 3.8.7, Bruker GmbH, Germany).

### Statistical Analysis

Metaboanalyst, an online freely available web interface, was used to perform multivariate statistical analysis. The data was Pareto scaled to obtain a symmetrical Gaussian distribution before metabolomic data analysis using Metaboanalyst. Orthogonal projections to latent structures-discriminant analyses (OPLS-DA) were performed to study the data points' variations. R2 and Q2 values were used to evaluate the goodness of fit and predictive ability of the regression models. The model validation was done using 10-fold cross-validation. The Permutation test (n=100) was also applied for identifying the reliability of the model.

S - and VIP- plots obtained from the OPLS-DA analysis aided in identifying the potential biomarkers. The metabolites which led to significant variation and correlation within the data set were selected as markers. Thus, generating the list of metabolites that can be identified as potential disease biomarkers having statistical significance (that is, metabolites that had a VIP score >1, and p-value <0.05, FDR < 0.05, correlation > 0.4).

## Results

For this study, 157 serum samples were collected: pretreatment (n=60) and post-treatment (n=97). Post-treatment serum samples were collected on the day I (n=37), day III (n=29), and day V (n=29). The metabolic phenotype of patients under treatment during their course of stay in the hospital was analyzed.

### Treatment affects the metabolome of patients with septic shock

**Figure 1:**
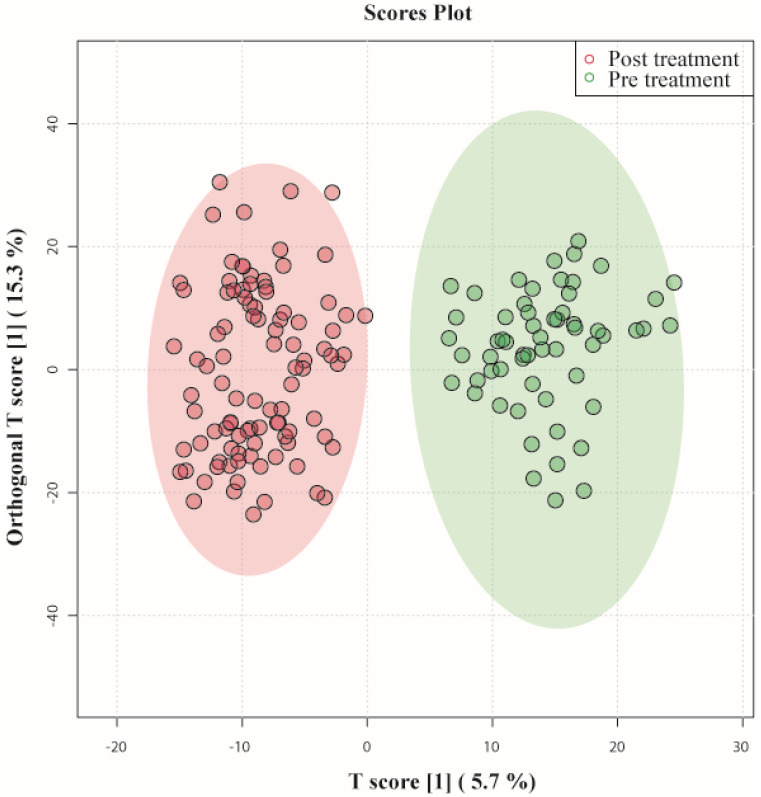
OPLS-DA score plots derived from 1D CPMG 1H NMR spectra of serum samples of patients with septic shock pre-treatment and post-treatment.

To systematically analyze the metabolic journey of patients with septic shock, OPLS- DA was performed on serum samples collected at the time of admission of patients (pretreatment) and post-treatment. In Figure 1 OPLS-DA showed that the two clusters are quite distinct from each other, illustrating a metabolic dissimilarity between the two groups. The OPLS-DA model generated had R2Y = 0.88 and Q2 = 0.78.

This indicates that both models were not overfitted, had good predictive power, and had a satisfactory fit. Multivariate analyses previously mentioned were used to identify the metabolites that showed a disparity between the two groups.

**Figure 2:**
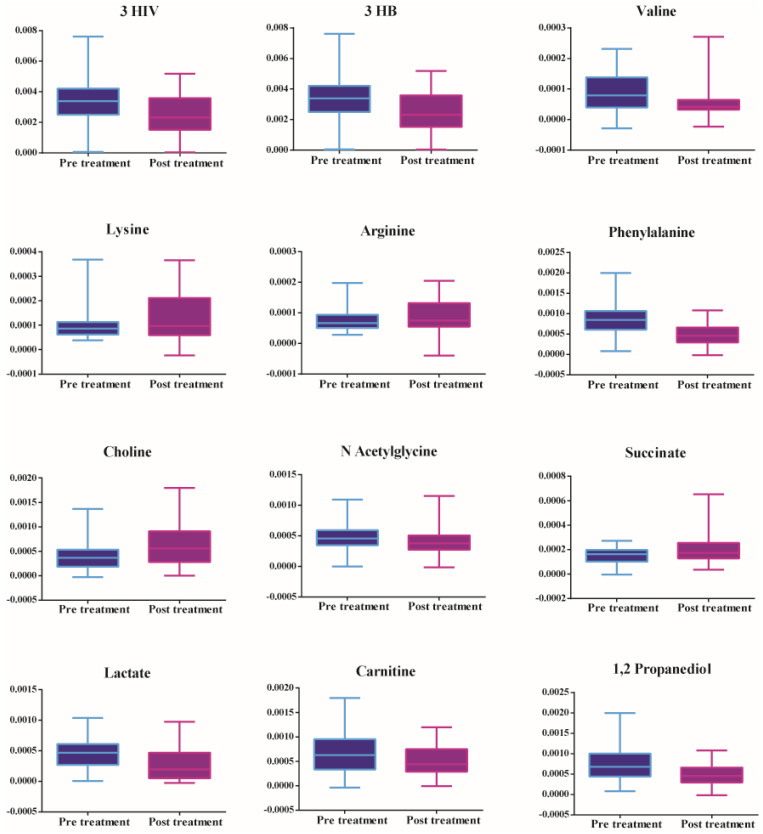
Significant metabolites between serum samples collected pre-treatment and post-treatment of patients with septic shock.

Figure 2 shows that there is an increase in amino acids (valine), ketone bodies (3 hydroxybutyrate and 3 hydroxyisovalerate), energy-related metabolites (lactate), mitochondrial β fatty acid oxidation (carnitine), and phenylalanine, decrease in amino acids (lysine and arginine), TCA cycle intermediates (succinate) and choline in patients prior to treatment. Table 3 shows the metabolites leading to the disparity between the two groups identified by multivariate statistics previously mentioned.

### Time-dependent metabolic changes occurring in septic shock patients during the course of treatment

**Figure 3:**
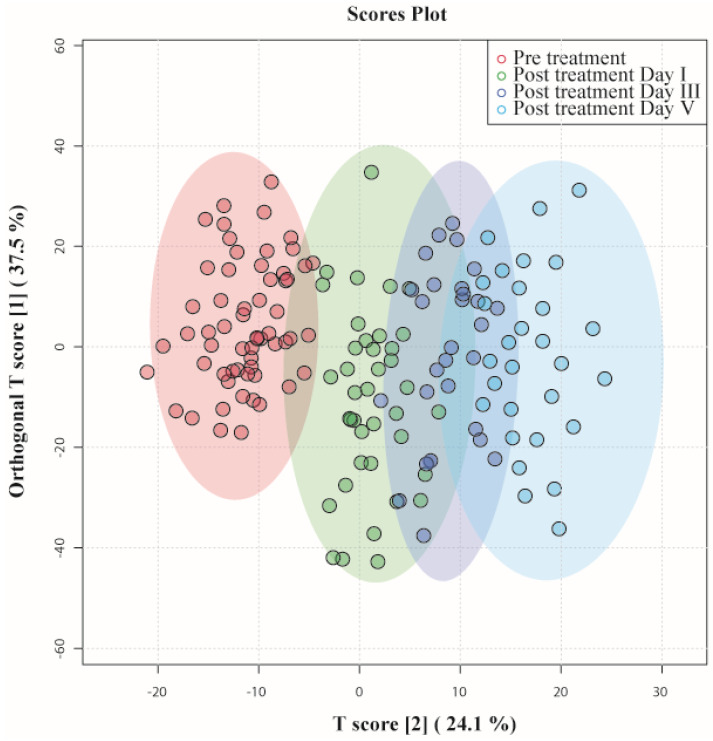
OPLS-DA score plots derived from 1D CPMG ^1^H NMR spectra of serum samples of patients with septic shock pre-treatment and post-treatment day I, day III, and day V.

OPLS - DA identified patients according to the time at which their samples had been collected and clustered them accordingly, as shown in Figure 3. Data points of day III and day V were far from pre-treatment, indicating that they had recovered from septic shock. On the one hand, there was a slight overlap between pretreatment and day I of post-treatment; their metabolic profiles had not evolved from the disease condition. On the other hand, certain data points of post-treatment day I overlap with day III, indicating that those patients' metabotypes have progressed from their previous day. The OPLS-DA model generated had R2Y = 0.70 and Q2 = 0.64.

**Figure 4:**
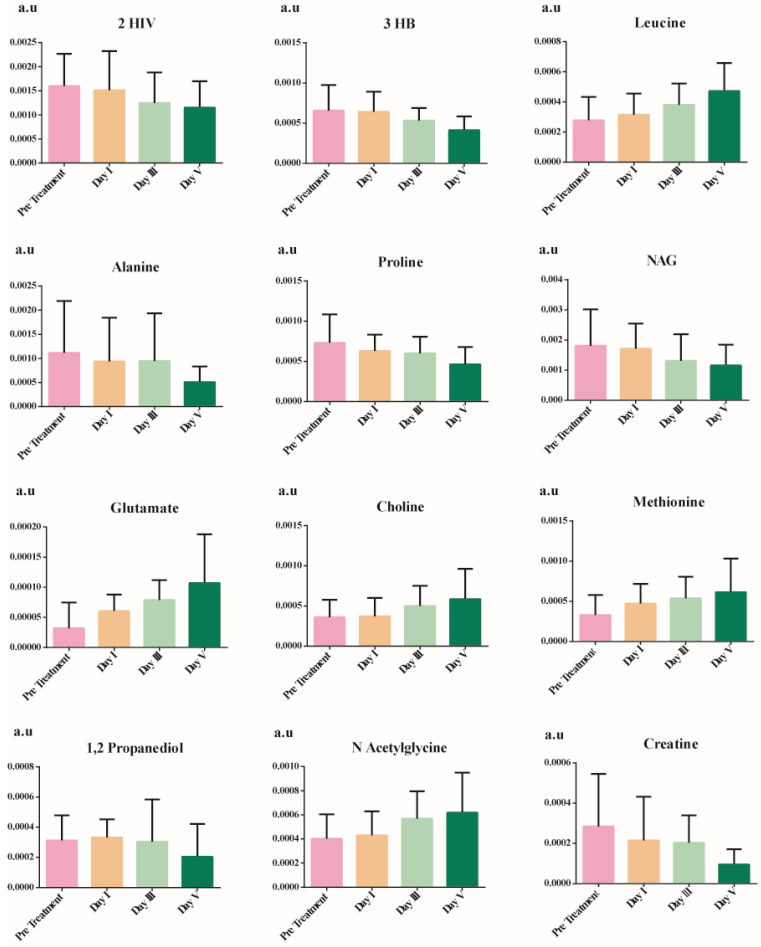
Significant metabolites showing time and phenotype-dependent changes between pre-treatment serum samples, post-treatment day I, day III, and day V.

The discriminatory metabolites were chosen based on their contribution to the variation and correlation within the data set. Additional *p*-value adjustments for multiple metabolites were carried out using Benjamini and Hochberg's (1995) false discovery rate (FDR) adjustment (Bonferroni Correction). Table 4 shows the list of potential discriminatory metabolites. Figure 4 shows a decrease in the levels of several amino acids (alanine and proline), ketone bodies, and NAG, whereas leucine, aspartate, methionine, and choline increase.

### Time-dependent metabolic changes occurring in septic shock survivors and non-survivor patients

**Figure 5:**
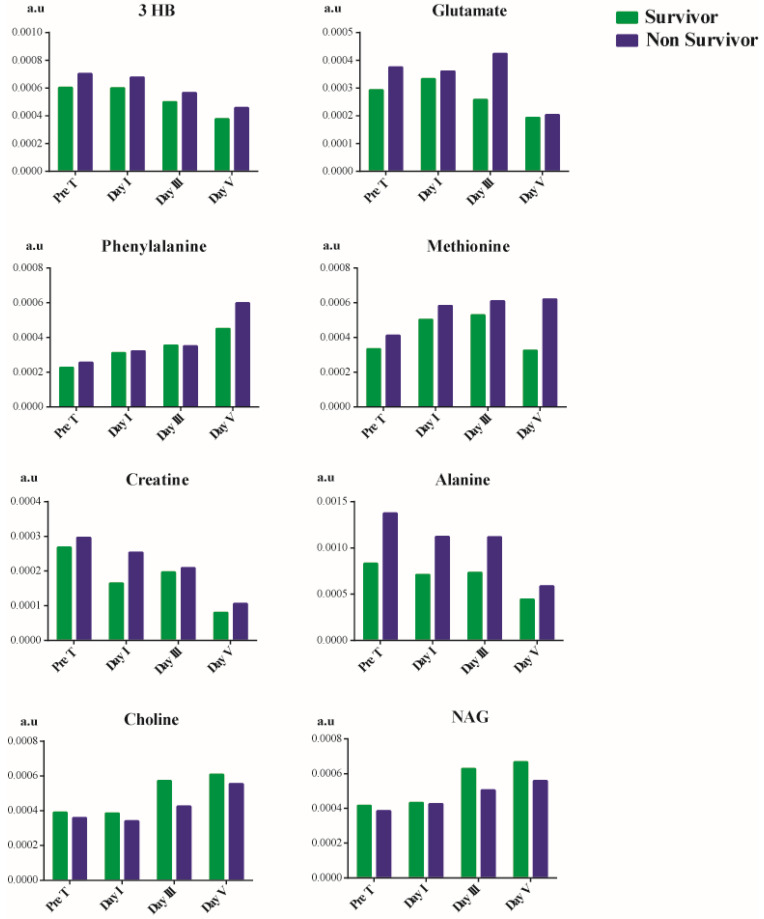
Metabolites showing variation from pre-treatment, post-treatment day I, day III, and day V between survivors and non-survivors of septic shock.

Due to the changes observed in the trend as previously mentioned, we explored any trend in metabolites of patients based on their mortality, as shown in Figure 5. Choline and NAG showed a decline in their concentration in septic shock non-survivors. In contrast, there was an overall increase in the concentration of 3 hydroxybutyrate, methionine, alanine, glutamate, phenylalanine, and creatine. The concentration of 3 hydroxybutyrate reduced considerably after treatment but was still higher than that of the survivors. The treatment had no effect in reducing the phenylalanine concentration. We observed a gradual increase in its concentration as the patient's hospital stay increased in survivors and non-survivors. In the case of creatine, the creatine concentration does decrease from before treatment till day V after treatment. Still, non-survivors had a higher concentration than survivors on day V. Treatment aid in reducing the concentration of 3 hydroxybutyrate, creatine, glutamate, and alanine in patients but is not significant enough to prevent mortality. In contrast, treatment was found to be ineffective in the case of phenylalanine and methionine.

## Discussion

This study aimed to investigate the impact of treatment on the serum metabolome of patients with septic shock and identify possible metabolic hotspots involved in the recovery of septic shock. The study also highlights the metabolic fingerprint of patients undergoing treatment during their stay in the hospital. The study was designed to focus on the metabotypes involved in the recovery of patients with septic shock. The study gave the metabolic biosignature of the patient's recovery. Analyzing serum samples of patients prior to treatment and throughout the three-day points (the day I, day III, and day V) of treatment allowed us to generate a metabolic journey of the course of patients' recovery.

### The pre-treatment period

Prior to treatment commencement, the metabolic profile of patients shows the characteristics of this heterogeneous disease, septic shock. In the state of infection, the increased energy requirements are obtained from breaking down fatty acids, which results in the elevation of ketone bodies (3 hydroxybutyrate and 3 hydroxyisovalerate) 32,33. Another cornerstone of septic shock is the resultant oxidative stress, which results in elevated anaerobic respiration, leading to elevated lactate in the serum 34,35. There is differential expression of amino acids in the serum of patients with septic shock; certain amino acids are elevated (valine), whereas amino acids (lysine and arginine) are declined in septic shock. Lysine plays an active role in immune response activity: reduces the tumor necrosis factor, interleukin -8, and macrophage inhibitory factor levels 36,37. However, since there is an imbalanced immune response in septic shock, it declines lysine in serum. Thus, as the patient undergoes treatment, it results in elevation of lysine resulting in an improving immune response.

Along with lysine, arginine also shows a decline in concentration prior to treatment. Arginase, an enzyme responsible for arginine catabolism to urea and ornithine, is up-regulated in septic shock 38,39. Thus, the resultant low level of arginine prior to treatment. The elevated valine 40 and phenylalanine 41 in serum are due to protein catabolism caused due to septic shock. Carnitine plays an important role in fatty acid oxidation in the form of a shuttle system in mitochondria 42. The low levels of succinate also signify mitochondrial dysfunction in septic shock patients prior to treatment. Succinate plays an important role in oxidative phosphorylation since septic shock leads to oxidative stress, thereby leading to suppression of oxidative metabolism and thus reduced succinate in serum 43. Their decline shows that septic shock leads to mitochondrial dysfunction. Choline is an important precursor of acetylcholine, and its decline affects the anti-inflammatory response 44, causing uncontrolled immune balance.

### The post-treatment period

Reacquiring homeostasis is an energy-challenging process involving various metabolites, as shown in Table 3. This study shows the complex changes that occur during the course of patient recovery from septic shock. The study unfolds the metabolic fingerprint of patients undergoing recovery, illustrating the reduction of ketone bodies and balancing amino acids and certain lipids to homeostasis.

One of the most prominent changes observed was the reduction in the level of ketone bodies from day I to day V of the patient under treatment. This is due to the body-switching to aerobic oxidation 45. Leucine is an essential amino acid required for the building block of proteins. Low levels of leucine indicate malnourishment; hence, as there is a progression towards recovery in patients, the body is rebuilding itself and thus increasing the concentration of leucine in serum. There is a gradual decrease in the level of alanine during the recovery of septic shock patients indicative of repaired glucose alanine cycle 46. Proline is recognized as a stress substrate during inflammation 47, hence gradually reducing concentration from the day I to day V of patient recovery. Glutamate plays a central role in the metabolism of the liver 48; as the patient is recovering, liver functions are improving, thus gradually elevating its concentrations. Another liver function test is aspartate aminotransferase which requires aspartate or glutamate as substrate, and as the patient progresses, the liver is improving 49. Thus, aspartate aminotransferase is efficient and hence increases in its substrate. Lastly, choline is shown to elevate gradually in the recovery from septic shock to bring back homeostasis in the immune response.

### Treatment effects on concentration of metabolites in patients based upon mortality

In non-survivors, there was an increase in the concentration of 3 hydroxybutyrate, creatine, glutamate, alanine, methionine, and phenylalanine. On the other hand, the concentration of choline and NAG showed a decline in concentration between survivors and non-survivor during the patient's hospital stay.

Treatment aided in reducing the concentration of 3 hydroxybutyrate, creatine, glutamate, and alanine but could not manage phenylalanine and methionine. Phenylalanine 50 and methionine 51 are the results of oxidative stress. This indicates that the treatment method is unable to target the oxidative state of the patients which could be a major role in leading to mortality in patients, even though there were improvements in other metabolites concentration.

## Conclusion

Various limitations need to be acknowledged. Though our study is of a decent sample size, it is still relatively small. The results need to be validated in a larger sample size. This study attempts to provide an insight into the metabolic signatures of early recovery of septic shock patients. This was our objective to enhance our understanding of the mechanistic underpinnings of septic shock. This is the first metabonomics study demonstrating the link between pre-treatment and post-treatment time-dependent metabolite changes in patients with septic shock. This type of metabotyping may help clinicians by monitoring therapeutics.

The results of this study can be considered the future of therapeutics, especially for septic shock. The metabolites identified can be used in therapy management with the potential for efficient monitoring of treatment by assessing the response of patients based upon the metabolites. It could benefit the patients undergoing septic shock treatment by assessing their response to treatment and giving an option to clinicians to alter the treatment plan according to patients' responses paving way for personalized medicine.

## Figures and Tables

**Table 1:** Tabular form of the consent obtained from the patients with septic shock

Tabular description of the details of the consent form	
Description of Clinical Investigation	This study involves the research for metabolic biomarkers of sepsis and septic shock. As long as the subject is admitted in ICU with sepsis and septic shock the serum samples would be collected for research purpose. An additional 2 ml of blood would be collected along with the routine blood collection done for clinical purpose in the hospital.
Risks and Discomforts	There is little reasonably foreseeable risks or discomforts to the subject The needle stick may hurt. There is a small risk of bruising and fainting.
Benefits	This research might not be a direct benefit for the subject at present but would contribute in enriching the data pool of biomarkers for sepsis and septic shock.
Confidentiality	An assurance of confidentiality of records identifying the subject will be maintained.
Contacts	Person of contact (PI of the research project) can be contacted for answers to pertinent questions about the research and research subjects' rights.
Voluntary Participation	A statement that participation is voluntary, that refusal to participate will involve no penalty or loss of benefits to which the subject is otherwise entitled, and that the subject may discontinue participation at any time without penalty or loss of benefits to which the subject is otherwise entitled.

**Table 2:** Demographic and clinical characteristics of the subjects enrolled in the study

Clinical Characteristics of patients in Septic Shock				
Variable	Pre-Treatment	Post-Treatment		
		Day I	Day III	Day V
No of patients	60	37	29	29
Sex (M/F)	29/31	23/14	20/9	20/9
Age	45.84±2.41	43±2.41	41.33±2.33	41.33±2.33
SOFA	17.5±0.50	13±0.7	12±1.2	8.5±0.50
TLC (x109/l)	18.5±1.39	14.3±1.25	11.5±1.02	10.5±1.2
Platelets (x109/l)	164.6±5.54	169±4.53	171±4.05	177±3.25
Serum Creatinine (mg/dl)	2.5±0.29	2.1±0.5	1.9±0.4	1.65±0.2
Serum Bilirubin(mg/dl)	2.19±1.10	1.7±1.05	1.5±1.2	1.3±0.5
PCT (ng/ml)	8.5±1.66	6.2±1.4	5.6±1.2	3±0.23

**Table 3:** List of significant small metabolites obtained between pre-treatment and post-treatment of patients with septic shock, identified using correlations (corr), VIP (variables importance of projection) obtained by the discriminant analysis, p-values adjusted with Bonferroni correction, FDR (false discovery rate)

S.No.	Name	Spectral location (ppm)	corr	p value	FDR	VIP
1	2-Hydroxyisovaleric acid	0.839	-0.307	0.007	0.004	1.014
2	Leucine	0.961, 0.972	0.277	0.003	0.02	1.045
3	Isobutyric acid	1.067	-0.172	0.036	0.005	1.100
4	Valine	0.996	-0.201	0.026	0.006	1.124
5	3-Hydroxybutyric acid	1.204	-0.331	0.000	<0.001	1.195
6	3 Hydroxyisovaleric acid	1.274	-0.212	0.007	<0.001	1.272
7	Lactate	1.332	-0.299	0.044	<0.001	1.069
8	Threonine	1.337	0.078	0.029	0.002	1.187
9	Lysine	1.452	0.272	0.012	0.003	1.285
10	Arginine	1.664	0.176	0.038	0.01	1.309
11	Methionine	2.140	-0.292	0.008	0.01	1.108
12	Succinic acid	2.415	0.228	0.001	0.007	1.040
13	Creatine	3.040	-0.277	0.010	0.01	1.004
14	Acetylcarnitine	3.201	-0.214	0.015	<0.001	1.021
15	Choline	3.209	0.356	0.01	<0.001	1.076
16	N-acetylglycine	3.751	-0.190	0.017	0.02	1.084
17	Phenylalanine	7.333	-0.344	<0.001	0.001	1.503
18	Carnitine	3.233	-0.118	<0.001	<0.001	1.453
19	1,2-propanediol	1.146	-0.257	0.004	<0.001	1.052

The cut off criterion being correlation >0.4, VIP >1, p-value < 0.05, FDR <0.05.

**Table 4:** List of significant small metabolites obtained between the pre- treatment and day I, day III, day V patients with treatment septic shock in ICU, identified using correlations(corr), VIP (variables importance of projection) obtained by the discriminant analysis

S.No.	Metabolite	Spectral location (ppm)	corr	p value	FDR	VIP
1	2-Hydroxyisovaleric acid	0.839	0.241	0.001	0.002	1.031
2	Isoleucine	0.944	-0.357	<0.001	<0.001	1.296
3	Leucine	0.961, 0.972	-0.331	0.004	0.007	1.329
4	2-Aminobutyric acid	0.984	0.260	0.004	0.007	1.125
5	Isobutyric acid	1.067	0.347	<0.001	<0.001	1.221
6	1,2-propanediol	1.146	0.505	<0.001	<0.001	2.283
7	3-Hydroxybutyric acid	1.204	0.252	<0.001	<0.001	1.032
8	Lactate	1.332	0.247	<0.001	0.001	2.578
9	Alanine	1.485	0.088	0.006	0.01	1.531
10	Proline	4.138	0.373	0.0001	0.01	1.123
11	NAG	2.02	-0.354	0.001	0.01	1.198
12	Glutamate	2.130	0.282	<0.001	<0.001	1.282
13	Acetone	2.236	0.213	0.01	0.02	6.831
14	Aspartic acid	2.675, 2.697	-0.518	<0.001	<0.001	1.151
15	Creatine	3.040	-0.372	<0.001	0.001	1.258
16	Phenylalanine	7.333	0.342	<0.001	<0.001	1.825
17	Acetylcarnitine	3.201	-0.282	<0.001	<0.001	1.691
18	Carnitine	3.233	-0.218	<0.001	<0.001	1.656
19	Methanol	3.362	-0.279	<0.001	<0.001	1.334
20	Choline	3.209	-0.275	<0.001	<0.001	1.068
21	Methionine	2.140	-0.392	<0.001	<0.001	1.438

p-values adjusted with Bonferroni correction, FDR (false discovery rate). The cut off criterion being correlation >0.4, VIP >1, p-value < 0.05, FDR <0.05. NAG- N- Acetylglucosamine.
